# Postpartum Bilateral Subdural Hematomas: A Case Report

**DOI:** 10.7759/cureus.11191

**Published:** 2020-10-27

**Authors:** Rachel E Bridwell, Neil P Larson, Mandie Baker, Amber Cibrario, Joshua Oliver

**Affiliations:** 1 Emergency Medicine, Brooke Army Medical Center, Fort Sam Houston, USA; 2 Emergency Medicine, Greater San Antonio Emergency Physicians, San Antonio, USA

**Keywords:** post dural puncture headache, post-partum headache, subdural hematoma

## Abstract

Postpartum headache is a common emergency department (ED) complaint in women following delivery. Though the majority of these headaches are due to primary headache disorders or postdural puncture headaches, life-threatening complications can occur. Both postpartum pre-eclampsia can occur as well as hypercoagulable and vascular disorders including stroke, venous sinus thrombosis (VST), and reversible cerebral vasoconstrictive syndrome. With the increasing use of epidurals for intrapartum analgesia, rare, dangerous complications can present in a similar fashion. The authors present a persistent postpartum headache secondary to bilateral subdural hematomas (SDH) from epidural induced intracranial hypotension.

## Introduction

Postpartum headaches are a common complaint, affecting 40% of postpartum patients [1]. Though the vast majority are caused by primary headache disorders, 4.7% are due to postdural puncture etiologies [1]. As this population is at particular risk for hypertensive and hypercoagulable disorders, other emergent considerations for postpartum headaches in the emergency department (ED) should be considered; these include postpartum pre-eclampsia, cerebral vascular accidents (CVA), venous sinus thrombosis (VST), and reversible cerebral vasoconstrictive syndrome [2-4]. Subdural hematomas (SDH) secondary to epidural placement is a rare but serious etiology of postpartum headache, with an estimated incidence of one in 500,000 intrapartum epidural anesthesia placements [5]. The authors present a case of persistent postpartum headache with bilateral SDH secondary to intracranial hypotension from epidural placement.

## Case presentation

**Figure 1:**
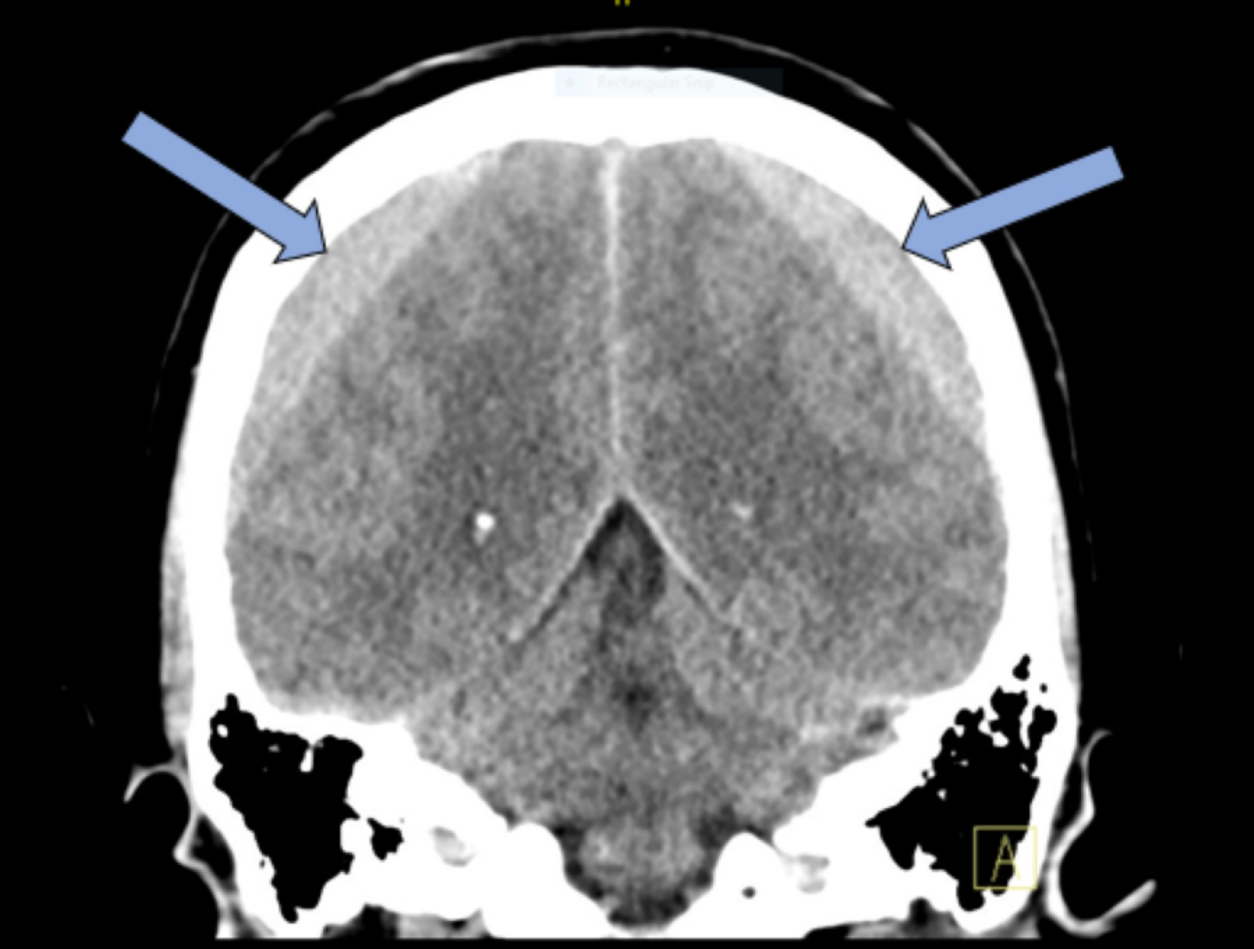
Coronal slice of a non-contrast head computed tomography demonstrating bilateral symmetric subacute subdural hematomas (blue arrows) with sulci effacement.

**Figure 2:**
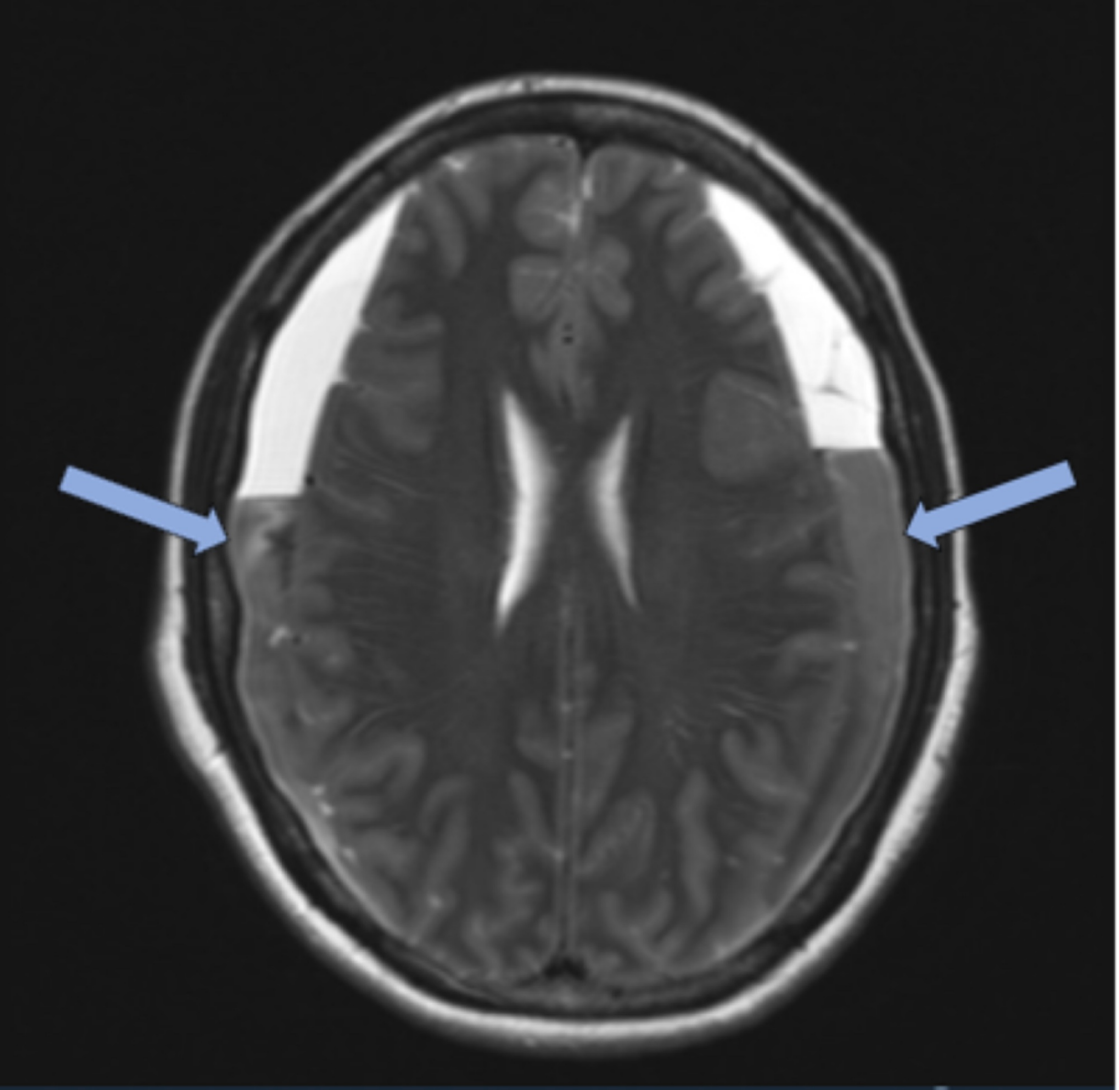
Axial slice of T2 magnetic resonance imaging demonstrating bilateral subacute subdural hematomas (blue arrows) with confirmed intracranial hypotension from cerebral spinal fluid leak.

A 35-year-old gravida 2 para 1 patient presented three weeks postpartum to the ED with persistent headache starting two days after delivery. She had an uncomplicated spontaneous vaginal delivery with epidural anesthesia requiring multiple attempts to place. She denied a history of gestational hypertension or pre-eclampsia. This bilateral frontotemporal headache with light sensitivity was refractory to acetaminophen-oxycodone and ibuprofen as well as a blood patch. On arrival, the patient's vital signs were a blood pressure of 140/86 mmHg, heart rate of 84 beats per minute, respiratory rate of 18 breaths per minute, temperature of 98.0 degrees Fahrenheit, and an oxygen saturation of 100% on room air. Her physical exam was unremarkable without lower extremity edema or focal neurologic deficits. A laboratory assessment revealed normal complete blood count, coagulation studies, fibrinogen, comprehensive metabolic panel, urinalysis, and a negative severe acute respiratory syndrome coronavirus-2 (SARS-CoV-2) test. Computed tomography of the head revealed symmetric bilateral subacute SDHs (Figure 1). Neurosurgery was consulted who requested magnetic resonance imaging of the brain, revealing subacute bilateral hematomas, which were secondary to intracranial hypotension from epidural placement (Figure 2). The patient was admitted to the intensive care unit without change in her clinical status. She was discharged on hospital day three with outpatient neurosurgical follow up.

## Discussion

Postpartum headache can be a presenting symptom of a variety of etiologies, ranging from primary headache and postdural headache disorder to CVA, VST, and the rare but life-threatening SDHs. Based on recent estimates, fewer than 100 cases have been reported [6]. While inadvertent dural puncture portends an odds ratio of 6.36 for postpartum headache, this case highlights a very rare complication from a common intrapartum procedure [1].

These patients will complain of headaches, usually presenting within the first four weeks, though cases have been documented as early as four hours and as late as far as 29 weeks postpartum [7]. Postpartum SDH should be considered in patients who have a postdural headache lasting greater than one week and refractory to a blood patch, a headache that changes from postural to non-postural, or in those who develop a focal neurologic deficit [7]. The most common presenting symptom is headache though nausea, vomiting, and altered mental status occur in one-third of cases [7-9]. While the majority of patients have a normal physical exam, approximately 25% will have a focal neurologic deficit [7-9]. An additional 20% will have diplopia or visual changes and 13% will demonstrate dysarthria or aphasia [7-9]. Interestingly, one case of postpartum SDH presented with neck stiffness but without headache, though this was after an elective cesarean delivery due to an unruptured cerebral aneurysm in the delivering mother [10].

While many patients will have unremarkable laboratory evaluation, platelets, fibrinogen, liver function tests, and urinalysis may identify hemolysis with elevated liver enzymes and low platelets (HELLP) and pre-eclampsia; additionally, patients with coagulopathy and thrombocytopenia are at increase risk of postpartum SDH [11]. Computed tomography is the best initial imaging modality in these postpartum cases, showing SDHs, which tend to be bilateral [12]. MRI may demonstrate chronic small SDHs, better evaluate concomitant spinal epidural hematomas, as well as help parse out this diagnosis from other diagnoses such as reversible cerebral vasoconstrictive syndrome [13].

The development of SDHs following epidural access is thought to be secondary to intraspinal and intracranial hypotension; in turn this causes ventricular collapse and caudal central nervous system migration, stretching the dura and tearing the bridging veins [10,14]. Early consultation to neurosurgery with admission to the intensive care unit for hourly neurologic assessments is key in managing these patients. While some cases can be treated with a blood patch, rebound intracranial hypertension can occur, requiring hematoma evacuation [15,16].

## Conclusions

Despite occurring in up to 40% of postpartum women, postpartum headaches range from benign primary headache disorders to life-threatening and rare etiologies. Emergency physicians should not only consider pre-eclampsia and hypercoagulable effects e.g. VST and CVA, but also the rare complications from epidurals, as evidenced by the SDHs presented in the above case. In persistent headaches greater than one week, refractory to initial appropriate treatments including blood patch for more common etiologies, emergency physicians should consider this rare complication and initiate advanced imaging.

## References

[1] Goldszmidt E, Kern R, Chaput A, Macarthur A (2005). The incidence and etiology of postpartum headaches: a prospective cohort study. Can J Anesth.

[2] Stanhope E, Foulds L, Sayed G, Goldmann U (2018). Diagnosing causes of headache within the postpartum period. J Obstet Gynaecol.

[3] Gao H, Yang BJ, Jin LP, Jia XF (2011). Predisposing factors, diagnosis, treatment and prognosis of cerebral venous thrombosis during pregnancy and postpartum: a case-control study [Article in Chinese]. Chin Med J.

[4] Allison SJ, Basit A, Mohd Hussein O, Ahmed RA (2013). Stroke in the postpartum period: a case study. J Clin Diagnostic Res.

[5] Scott DB, Hibbard BM (1990). Serious non-fatal complications associated with extradural block in obstetric practice. Br J Anaesth.

[6] Szeto V, Kosirog J, Eilbert W (2018). Intracranial subdural hematoma after epidural anesthesia: a case report and review of the literature. Int J Emerg Med.

[7] Amorim JA, Remígio DSCA, Filho OD, de Barros MAG, Carvalho VN, Valença MM (2010). Intracranial subdural hematoma post-spinal anesthesia: report of two cases and review of 33 cases in the literature [Article in English, Portuguese]. Rev Bras Anestesiol.

[8] Lim G, Zorn JM, Dong YJ, DeRenzo JS, Waters JH (2016). Subdural hematoma associated with labor epidural analgesia: a case series. Reg Anesth Pain Med.

[9] Cuypers V, Van De Velde M, Devroe S (2016). Intracranial subdural haematoma following neuraxial anaesthesia in the obstetric population: a literature review with analysis of 56 reported cases. Int J Obstet Anesth.

[10] Domoto S, Suzuki M, Suzuki S, Bito H (2018). Subdural hematoma after cesarean delivery without symptoms: a case report. JA Clin Rep.

[11] Zeidan A, Farhat O, Maaliki H, Baraka A (2006). Does postdural puncture headache left untreated lead to subdural hematoma? Case report and review of the literature. Int J Obstet Anesth.

[12] Cohen JE, Godes J, Morales B (1997). Postpartum bilateral subdural hematomas following spinal anesthesia: case report. Surg Neurol.

[13] Liu G, Lee A, Withanawasam N, Tara S (2020). Subdural hemorrhage post obstetric epidural: an MRI case report. Radiol Case Rep.

[14] Wilhelm S, Standl T (1997). Continuous spinal anesthesia vs. combined spinal-epidural anesthesia in emergency surgery. The combined spinal-epidural anesthesia technique does not offer an advantage of spinal anesthesia with a microcatheter [Article in German]. Anaesthesist.

[15] Hashizume K, Watanabe K, Kawaguchi M, Fujiwara A, Furuya H (2013). Evaluation on a clinical course of subdural hematoma in patients undergoing epidural blood patch for spontaneous cerebrospinal fluid leak. Clin Neurol Neurosurg.

[16] Davies JM, Murphy A, Smith M, O’Sullivan G (2001). Subdural haematoma after dural puncture headache treated by epidural blood patch. Br J Anaesth.

